# Investigation of Structural Energy Absorption Performance in 3D-Printed Polymer (Tough 1500 Resin) Materials with Novel Multilayer Thin-Walled Sandwich Structures Inspired by Peano Space-Filling Curves

**DOI:** 10.3390/polym15204068

**Published:** 2023-10-12

**Authors:** Peng Lin, Zhiqiang Zhang, Yun Chen, Dayong Hu

**Affiliations:** 1 Department of Biomedical Engineering, College of Engineering, Shantou University, Shantou 515063, China; 21plin@stu.edu.cn (P.L.); 20wchen@stu.edu.cn (Y.C.); 2 Department of Aircraft Airworthiness Engineering, School of Transportation Science and Engineering, Beihang University, Beijing 100191, China; hudayong@buaa.edu.cn

**Keywords:** Peano space-filling curves, multilayer thin-walled sandwich structures, energy absorption, 3D-printed polymer materials, optimization

## Abstract

Inspired by Peano space-filling curves (PSCs), this study introduced the space-filling structure design concept to novel thin-walled sandwich structures and fabricated polymer samples by 3D printing technology. The crushing behaviors and energy absorption performance of the PSC multilayer thin-walled sandwich structures and the traditional serpentine space-filling curve (SSC) multilayer thin-walled sandwich structures were investigated using quasi-static compression experiments and numerical analysis. Taking the initial peak crushing force (*IPF*), specific energy absorption (*SEA*), and crushing force efficiency (*CFE*) as evaluation criteria, the effects of geometric parameters, including the curve order, layer height, septa thickness, and wall thickness, on energy absorption performance were comprehensively examined. The results indicated that the energy absorption capacity of the PSC structure was significantly enhanced due to its complex hierarchy. Specifically, the second-order PSC structure demonstrated a 53.2% increase in energy absorption compared to the second-order SSC structure, while the third-order PSC structure showed more than a six-fold increase in energy absorption compared to the third-order SSC structure. Furthermore, a multi-objective optimization method based on the response surface method and the NSGA-II algorithm were employed to optimize the wall thickness and layer height of the proposed novel PSC structures. The optimal solutions suggested that a reasonable wall thickness and layer height were two important factors for designing PSC structures with better energy absorption performance. The findings of this study provide an effective guide for using the space-filling concept with Peano curves for the design of a novel polymer thin-walled energy absorber with high energy absorption efficiency.

## 1. Introduction

Thin-walled structures have found extensive applications across various domains, including the automotive sector, aerospace, civil engineering, biomedicine, and more. This popularity stems from their remarkable attributes, such as extremely low weight, cost-effective manufacturing, and exceptional energy absorption efficiency [1,2,3,4,5,6,7,8,9,10,11,12]. Over the past few decades, conventional thin-walled tubes like circular tubes [13,14], triangular tubes [5,15], square tubes [16], and polygonal tubes [17,18] have been the subject of extensive research and investigation. However, simple tubes suffer from their limited design space, which makes it difficult to further improve their performance. Improving the mechanical properties of thin-walled structures has become a prominent research area within the field of lightweight energy absorption. Several novel thin-walled design strategies have been proposed and investigated, including various cross-sectional configurations [19,20,21,22], structural hierarchies [1,23], multi-cell tubes [7,24], gradient thicknesses [25,26,27,28,29,30,31], composite tubes [6,32], and form filling [4,8,33,34].

The utilization of multi-cell tubes has gained considerable attention in the pursuit of enhancing the crashworthiness of thin-walled structures, owing to their exceptional energy absorption characteristics [35,36]. Wierzbicki and Abramowicz delved into the folding mechanism of thin-walled structures and the crushing behavior of multi-corner columns. They established that the energy absorption performance of these structures was heavily influenced by the number of corner elements, and they successfully predicted the mean crushing force of multi-corner columns subjected to axial compression [37,38]. Kim introduced innovative multi-cell profiles featuring four square elements at the corners, resulting in significant improvements in crash energy absorption and weight efficiency when compared to conventional square box columns [39]. Chen and Wierzbicki introduced single-layer and double-layer partitions into hollow columns [40]. Through numerical simulations, it was found that the multi-cell tube could significantly enhance the compressive strength of the structure. The concept of hierarchy was incorporated into thin-walled circular tubes by Zhang et al. [41]. Their findings demonstrated that the crushing performance of these tubes could be significantly enhanced by substituting the original single-cell circular tube wall with a series of smaller tubes. Recently, the multi-cell approach has been demonstrated to improve the crashworthiness of structures. However, multi-cell tubes still have a broad design space.

More recently, the exploration of hierarchical structures found in biological tissues has sparked innovative approaches to designing high-performance energy absorbers [1,42,43]. Wu et al. introduced triangular, square, and pentagonal tree-like fractal structures, which exhibited significant potential for enhancing energy absorption properties when compared to single-walled structures [44]. Ha et al. took inspiration from nature and constructed a novel bio-inspired hierarchical multi-cell square (BHMS) tube, mimicking the gradient distribution of cell sizes found in biological structures like bones and bamboo. Compression simulations of different-order BHMS structures revealed that high-order BHMS tubes possessed substantially higher *SEA* capabilities compared to their low-order counterparts [45]. Drawing inspiration from the growth patterns and microstructures of plant stems, Gong et al. introduced a series of tubes with m parts and n layers (PmLnBTs). Their theoretical predictions and numerical analyses indicated that PmLnBTs exhibited superior crashworthiness performance when compared to traditional bi-tubular circular tubes [46]. Wang et al. [47] introduced an innovative thin-walled multi-cell tubular structure featuring a modified face-centered cubic (MFCS) cross-section, drawing inspiration from the unique characteristics of the glass sponge’s skeletal structure. When compared to conventional multi-cell tubes, the MCFS structure displayed micro-folding lobes with shorter wavelengths, leading to significantly improved energy absorption efficiency. Moreover, the mean crushing force of this MCFS structure approached that of an ideal energy absorber. Besides the above-mentioned tube structures, sandwich structures with thin-walled cores have also been heavily used for energy absorption. Thin-walled sandwich structures typically consist of stiff skin layers and corrugated core sheets, resulting in superior bending stiffness, strength, and *SEA* properties compared to solid monolithic construction [48,49]. Dayyani et al. categorized sandwich panels into three distinct groups: curved corrugated core sandwich panels, bi-directional corrugated core sandwich panels, and hierarchical corrugated core sandwich panels [50]. Notably, the use of curved corrugated sheets, including triangular and trapezoidal shapes, as cores for sandwich panels has been the subject of extensive research and investigation [51,52,53]. Li et al. [54] employed 3D printing technology to fabricate Grid-shaped, V-shaped, and U-shaped corrugated sandwich structures, allowing for a comparison of the mechanical properties of various shapes and layers. Their findings showed that the deformation modes of these sandwich structures were influenced by the load-carrying path and the relative mechanical properties between interlayers and cores. Yang et al. introduced an innovative lightweight bi-directionally sinusoidal corrugated core sandwich panel inspired by mantis shrimp structures [55]. Their research demonstrated that bi-directional corrugated core sandwich panels exhibited significantly enhanced crashworthiness compared to conventional triangular and sinusoidal corrugated sandwich panels. In order to enhance the energy absorption capacity of thin-walled core sandwich panels, Kooistra et al. [56] introduced the concept of hierarchical corrugated core sandwich panels in their study [56]. They conducted a comparative analysis examining the transverse compression and shear collapse mechanisms of first-order and second-order corrugated truss structures. Their research findings revealed that the strength of second-order trusses was approximately ten times greater than that of first-order trusses with the same relative density. This substantial increase in strength contributed significantly to boosting the *SEA* of the hierarchical corrugated core sandwich panel.

In order to improve energy absorption capabilities, many researchers have also proposed multilayer thin-walled sandwich structures. Energy absorption in these sandwich panels demonstrates an increase when the number of layers increases. Notably, there has been substantial interest in the field of crashworthiness research in structural design methods that draw inspiration from biological multilayer structures. This is due to their ability to generate unique geometric shapes and enhance energy absorption efficiency. For instance, the cuttlebone has a complex porous structure and mechanics with an asymmetric S-shaped wall structure connecting laminar septa, which helps it to withstand significant hydrostatic pressure in deep-sea environments. Polymers are frequently employed as raw materials for the 3D printing of high-precision structures, owing to their inherent plasticity and resilience. Consequently, numerous researchers have utilized 3D-printed polymer specimens to simulate and analyze the mechanical properties of complex structures [57,58]. Mao et al. [59] employed polymeric materials to manufacture structures resembling cuttlebones that exhibited superior strength and energy absorption capabilities compared to octet-truss lattices, as well as traditional polymer and metal foams. In addition, Wu et al. [60] optimized and designed a new elliptical corrugated cuttlebone-like multilayer structure based on the sinusoidally corrugated cuttlebone-like multilayer structure and subsequently utilized 3D printing technology with a C-UV9400E photosensitive resin to manufacture specimens for mechanical analysis, which significantly improved the compressive and shear capacities. The multilayer structure of the cuttlebone, interconnected by thin-walled structures, greatly enhances the cuttlefish’s survival ability in the deep sea and also provides inspiration for the design of new multilayer sandwich structures.

As already mentioned, well-designed structures with complex geometries, such as hierarchical multi-cell tubes, hierarchical corrugated core sandwich panels, and cuttlebone-like multilayer sandwiches, have better crashworthiness performance than conventional thin-walled structures according to all evaluation criteria. From a geometric perspective, the absorbing energy of a thin-walled sandwich structure can be regulated by changing the filling materials in the core space. Theoretically, space-filling curves (SFCs) are characterized by a unique property: that is, a specific space can be completely filled with a continuous curve of infinite length after an infinite number of iterations. Peano space-filling curves, as the most well-known SFCs, have the capability to fill a defined design space with an infinite length of curves. In the context of thin-walled sandwich structures inspired by Peano space-filling curves and with a specified relative density, it is possible to fill the cross-sections of their cores with infinite space-filling curves by progressively increasing the level of fractal hierarchy. Therefore, thin-walled sandwich structures with cross-sectional configurations of Peano space-filling curves could have great potential for offering excellent energy absorption capacity in the axial loading direction. In addition, the linear continuity and highly fractal characteristics of Peano space-filling curves could give thin-walled sandwich structures many multifunctional advantages, which could meet the crashworthiness requirements of equipment such as supercapacitors, jet engine inlets, and heat exchangers [61,62,63]. However, Peano space-filling curves have rarely been used as the design topic for thin-walled sandwich structures. 

To fill the above gap, inspired by Peano space-filling curves, novel multilayer thin-walled sandwich structures were proposed and fabricated in this study. The paper is organized as follows: Section 2 presents the evolution process of SFCs and the fabrication of structures. Section 3 describes the experimental setups used to study the material mechanical properties and quasi-static axial crushing of SSC structures, as well as crashworthiness indicators and the finite element (FE) model setup. In Section 4, the results of numerical simulations of the second-order SFC structures are verified with experimental results. In this section, we also present an analysis and comparison of the energy absorption characteristics of two different types of SFC sandwich structures. Furthermore, we delve into the influence of geometric parameters on energy absorption performance. Finally, Section 5 explores the optimal configurations of PSC structures by establishing the response surface model. The conclusions are summarized in Section 6.

## 2. Models’ Description and Fabrication

### 2.1. Models’ Description

**Figure 1:**
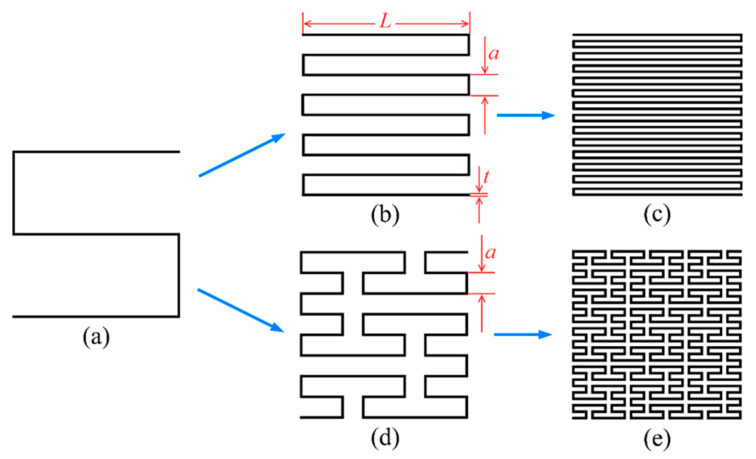
A sketch of two kinds of SFCs: (**a**) first-order space-filling curve; (**b**) second-order serpentine space-filling curve (SSC2); (**c**) third-order serpentine space-filling curve (SSC3); (**d**) second-order Peano space-filling curve (PSC2); (**e**) third-order Peano space-filling curve (PSC3).

Figure 1 illustrates the evolution process of two types of SFCs, both of which evolved from a first-order curve, as shown in Figure 1a. Figure 1b,c represent serpentine space-filling curves (SSCs), which become gradually denser in space when shortening the length of their short edges and increasing the number of curve bends. Figure 1d,e depict Peano space-filling curves (PSCs). The second-order Peano space-filling curve (PSC2) is shown in Figure 1d, which is formed by connecting nine first-order curves arranged according to certain rules. Similarly, the third-order Peano space-filling curve (PSC3) is formed by connecting nine PSC2 curves, as shown in Figure 1e.

**Figure 2:**
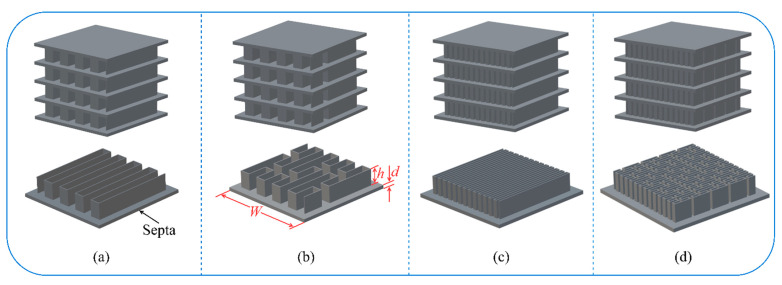
Multilayer thin-walled sandwich structures inspired by SFCs: (**a**) SSC2 structure; (**b**) PSC2 structure; (**c**) SSC3 structure; (**d**) PSC3 structure.

Inspired by the geometric shapes of SFCs, four multilayer thin-walled sandwich absorption structures were designed and fabricated, as depicted in Figure 2a–d. These structures consisted of four single-layer models, and each single-layer model comprised two components: a thin-walled structure with a height of h= 10 mm and a square septum with a thickness of d= 2 mm and a side length of W, which was 1.2 times the side length of the thin-walled structure. The geometric parameters of the thin-walled structure are described in Figure 1b, where t= 0.5 mm and L= 50 mm denote the thickness of the wall and the width of the structure. Additionally, the rectangular element width a of the second-order serpentine space-filling curve (SSC2) model in Figure 1b was equal to that of PSC2 in Figure 1d.

### 2.2. Specimen Fabrication

**Figure 3:**
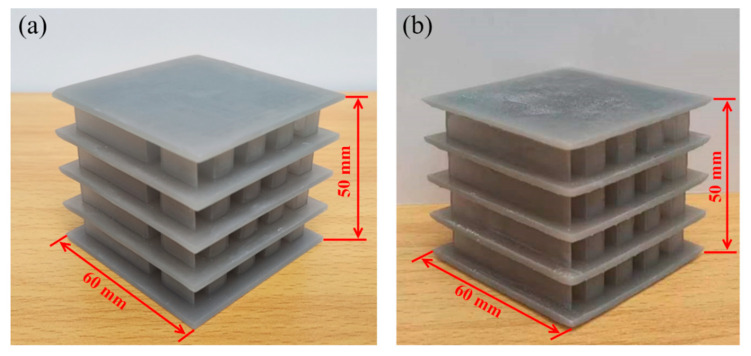
Specimens fabricated by 3D printing technique: (**a**) SSC2 specimen; (**b**) PSC2 specimen.

Common 3D printing technologies include selective laser sintering (SLS), fused filament fabrication (FFF), and stereolithography apparatus (SLA). SLS is often employed to process metal materials due to its simple manufacturing process, but it has long processing times and produces components with rough surfaces. FFF offers advantages such as low cost and high production efficiency, but the mechanical properties of the printed parts are relatively poor. Although introducing continuous fibers can enhance part strength, challenges like low processing accuracy and a rough surface finish still persist [64]. SLA is the most mature and widely applied technology in the field, known for advantages such as its fast forming speed, high processing accuracy, and excellent surface quality of components. In this study, the specimens SSC2 and PSC2 were fabricated using the SLA 3D printing technique with a Form 3+ printer (Formlabs, Somerville, Massachusetts, USA). The printing parameters used were as follows: formed platform volume of 14.5 mm × 14.5 mm × 18.5 mm, printing accuracy of 0.085 mm, layer thickness of 25–300 um, laser power of 250 mW, and printing temperature of 35 °C. Tough 1500 Resin, a polymer material with excellent strength and toughness, was selected as the printing material. After printing, the specimens were cleaned in 95% alcohol and then solidified for 40 min at 70 °C under UV light. Figure 3a,b depict the two fabricated specimens.

## 3. Experimental Setups and Numerical Model

### 3.1. Material Properties

**Figure 4:**
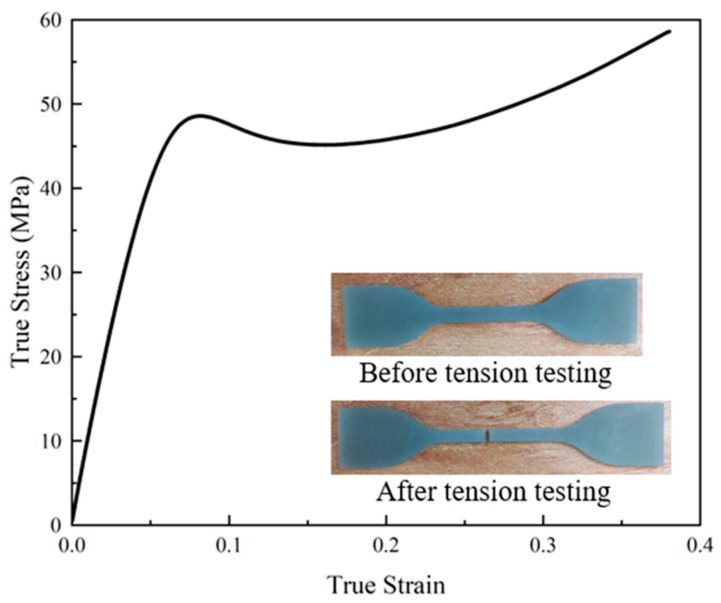
True stress–strain curve of Tough 1500 Resin tensile specimen.

To acquire dependable mechanical performance data for the material, standard tensile specimens were manufactured according to the ASTM Standard D412-16 [65], and the tensile test was conducted using a universal testing machine with a 20 kN capacity, maintaining a constant velocity of 5 mm/min. To ensure the accuracy of the test results, three tensile specimens were produced and tested, with the results demonstrating good repeatability. From the test, one specimen was selected to generate the true stress–strain curve of the material, which is presented in Figure 4. It is worth noting that the stress of conventional stress–strain behavior of metal materials usually increases until fracture after reaching the yield stress. However, when the standard tensile specimens fabricated with Tough 1500 Resin reached the yield stress, the stress value decreased, which increased the strain before the fracture of the tensile specimen, indicating that this polymer material had excellent plasticity and ductility. The primary mechanical properties of Tough 1500 Resin are as follows: density ρ=1210 $kg/m^{3}$, Young’s modulus E= 850 MPa, Poisson’s ratio ν= 0.35, and initial yield stress $\sigma_{y}=$ 48.58 MPa. It should be noted that in our finite element (FE) modeling, we did not account for the strain rate effect, as our simulations were focused on quasi-static loading conditions.

### 3.2. Axial Crushing Experiments

**Figure 5:**
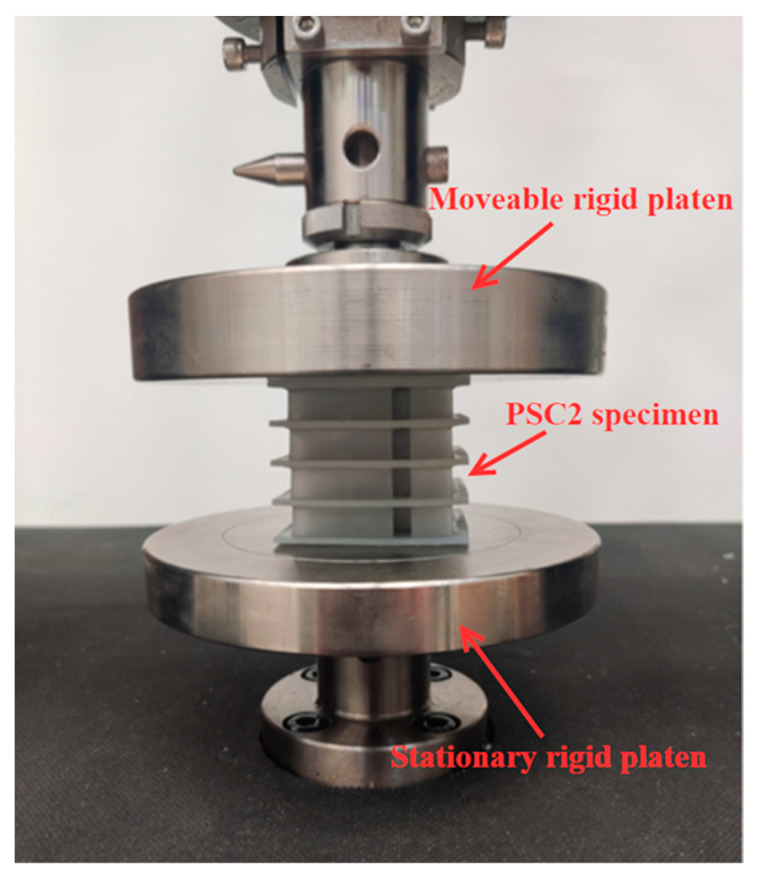
Experimental setup for axial compression test.

Figure 5 illustrates the results of axial crushing experiments aimed at investigating the energy absorption characteristics of the PSC2 structure. The experimental setup involved positioning the specimen between two rigid platens, with the lower platen being fixed and the upper platen being subjected to a constant speed of 5 mm/min. The complete crushing process of the specimens was captured on camera. Two identical tests were carried out to ensure the repeatability of the experiments.

Several crashworthiness indices were used to assess the mechanical performance as follows.

*IPF* is the maximum peak force that appears at the initial stage for the first time.

*EA* indicates the total energy absorption of the structure in the deformation process, and it can be obtained by the following expression:

(1) $$EA=\int_{0}^{\delta }F\left(x\right)dx$$

where F(x) represents the instantaneous crushing force, and δ is the densification compression displacement.

*SEA* indicates the energy absorbed per unit of mass of the structure:

(2) $$SEA=\frac{EA}{m}=\frac{\int_{0}^{\delta }F\left(x\right)dx}{m}$$

where m is the total mass of the structure.

*MCF* assesses the mean crushing force of the structure and is defined by:

(3) $$MCF=\frac{EA}{\delta }=\frac{\int_{0}^{\delta }F\left(x\right)dx}{\delta }$$

*CFE* is the crushing force efficiency, which can effectively measure the level of force fluctuation:

(4) $$CFE=\frac{MCF}{IPF}$$

### 3.3. Finite Element Simulation

**Figure 6:**
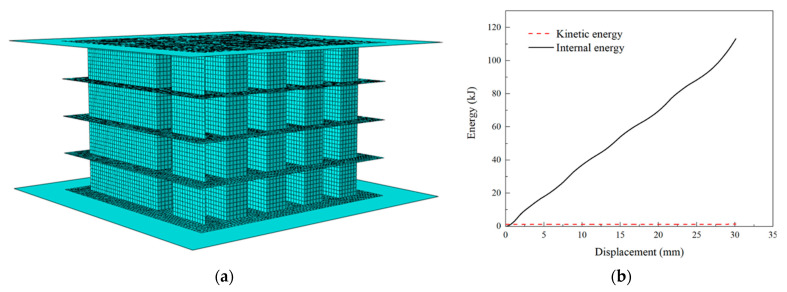
Finite element simulation setups: (**a**) FE model; (**b**) comparison of total kinetic and internal energy for the FE model.

**Figure A1:**
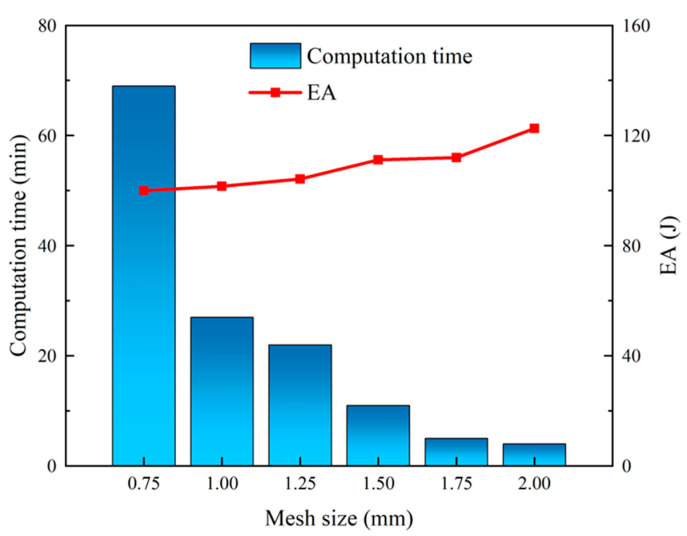
Convergence analysis results of the mesh size.

The numerical model utilized in this study was established using the commercial software ABAQUS 2021/Explicit. As shown in Figure 6a, the finite element (FE) model of PSC2 was sandwiched between two rigid platens. In order to simplify the numerical model and improve computation efficiency, the reduced integral shell element S4R node was implemented for meshing, while the discrete rigid element R3D4 was employed to model the two rigid platens. A sensitivity analysis was conducted on six different densities to determine an optimal mesh size. The results indicated that an element size of 1 mm × 1 mm could provide accurate results while maintaining a reasonable computation time, as presented in Figure A1. To simulate the loading process, a dynamic explicit analysis step was integrated. The general contact algorithm, along with the self-contact of the specimen, was applied to the specimen and two rigid platens. The friction coefficient between the model and rigid platens was set to 0.2. The lower platen was constrained with all of the degrees of freedom (DOFs), while the upper platen retained only the DOFs in the height direction. The velocity of the upper moveable platen was set at 0.5 m/s. As depicted in Figure 6b, the changes in the total kinetic energy and the total internal energy of the PSC2 model were recorded during the crushing process. The results indicated that the total kinetic energy was less than 0.1% of the total internal energy, suggesting that the scaled-up velocity could be applied in quasi-static loading scenarios.

## 4. Results and Discussion

### 4.1. Finite Element Simulation

**Figure 7:**
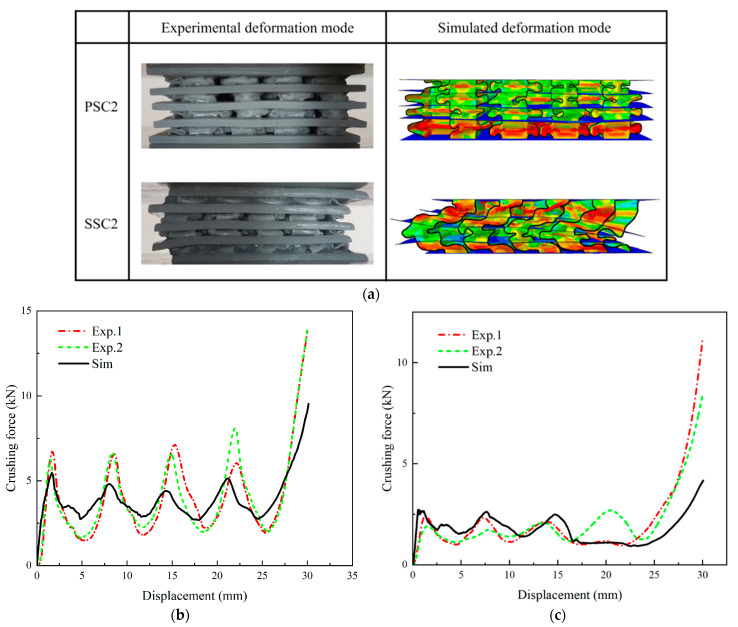
Comparison of experiment and FE simulation: (**a**) the final deformation in experiment and simulation; (**b**) crushing-force–displacement curves of PSC2 structures; (**c**) crushing-force–displacement curves of SSC2 structures.

The results of the quasi-static axial crushing experiment on the PSC2 and SSC2 structures are presented in Figure 7a. The layers in the PSC2 specimen were entirely compressed, and post-compression deformation analysis revealed that the primary energy dissipation mechanism was folding, resulting from the inward contraction or outward expansion of thin-walled structures within the specimen. However, the thin-walled core of the SSC2 structure tilted to one side during compression, and bending deformation also occurred on the septa. Figure 7a also shows the numerical simulation results, demonstrating that the two models exhibited the same deformation mode as observed in the experiments. The crushing-force–displacement curves of the PSC2 structure obtained from FE simulations and two axial crushing experiments showed similar trends, as shown in Figure 7b. Each curve had four peaks, with each peak value representing the crushing process of a single layer. Figure 7c illustrates the experimental and simulated force–displacement curves of the SSC2 structure, which exhibited similar variation trends. The comparison of *SEA* and *MCF* data between the experiments and simulations is shown in Table 1, indicating good consistency between the experimental and simulation results.

**Figure 8:**
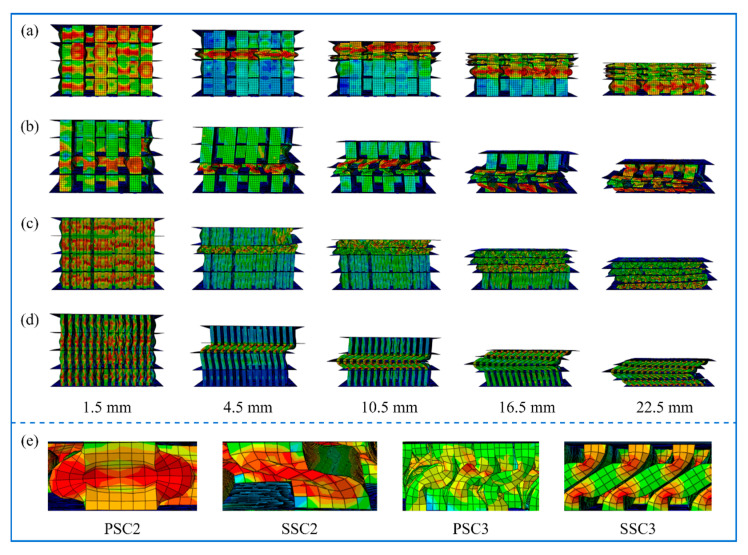
Numerical simulation results for four models at different crushing displacements: (**a**) PSC2 model; (**b**) SSC2 model; (**c**) PSC3 model; (**d**) SSC3 model; (**e**) the main deformation mode.

To further investigate the energy dissipation capacity of the PSC structure, the axial crushing performance of PSC3 and third-order serpentine SFC (SSC3) models was also studied. SSC2 and SSC3 models had the same curve length and rectangular element width as the PSC2 and PSC3 models, respectively. In addition, the third-order model had the same quality as the second-order model, with the wall thicknesses of the second and third models set at 0.5 mm and 0.18 mm, respectively. The numerical simulation results depicting the deformation behavior of the four models under quasi-static axial compression are presented in Figure 8. In Figure 8a,c, it can be observed that during the deformation of the second-order and third-order PSC structures, the thin-walled core folded along the force direction, while the four-layer thin-walled sandwich structure exhibited a progressive buckling mode. In contrast, Figure 8b,d show that the thin-walled core of the second and third-order SSC structures tilted to one side during the compression process, leading to an overall unstable deformation. Figure 8e illustrates the primary deformation modes of the four structural units. The PSC2 structure demonstrated outward expansion or inward contraction in the middle of the thin wall, exhibiting favorable energy dissipation characteristics. On the other hand, the SSC2 structure’s thin walls showed instability during the deformation process, resulting in a collapse to one side. Comparatively, the deformation modes of PSC3 and SSC3 structures were similar, with the thin walls bending in both the upper and lower parts. However, the folding degree of the PSC3 thin wall was more pronounced than that of the SSC3 thin wall, thereby rendering the PSC3 model stable and the SSC3 model unstable.

**Figure 9:**
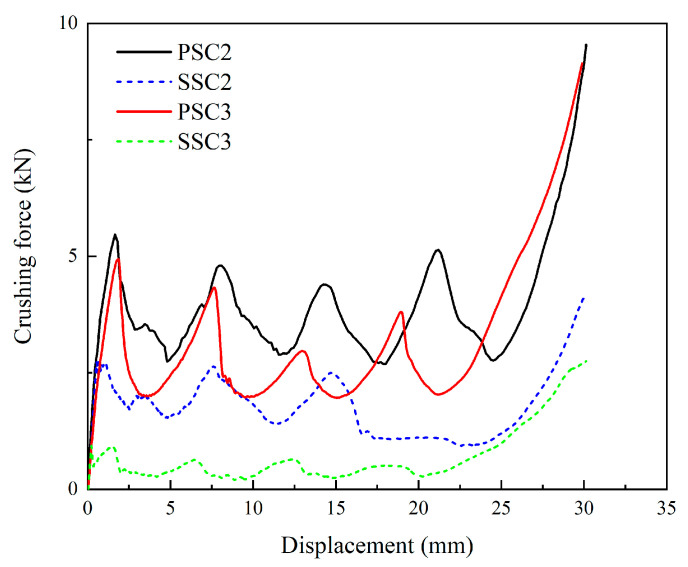
Crushing-force–displacement curves of two parts of comparison models.

Figure 9 illustrates the force–displacement curves of two parts of comparison models subjected to axial compression, and Table 2 compares the performance of the four models based on various indicators, including *IPF*, *MCF*, *SEA*, and *CFE*. As depicted in Figure 9, the crushing force of the PSC2 and PSC3 structures was significantly higher than that of their respective comparison models. Regarding energy absorption, the *EA* of the PSC2 model reached 101.22 J, which is 53.2% higher than that of the SSC2 model, and the PSC3 model exceeded its comparison model by over six-fold. Therefore, the implementation of the rectangular element in the PSC structure significantly improved the crashworthiness of the model.

### 4.2. Influence of Different Parameters

For the sake of further investigating the effects of the crushing response and energy absorption characteristics of PSC structures with different parameters, this section examines several different parameters, including layer height, septa thickness, and wall thickness. The FE model established in Section 3.3 was used, with the other parameters remaining constant while one parameter was changed.

#### 4.2.1. Effect of Layer Height

To investigate the impact of the layer height on the energy absorption capacity of PSC2 structures, FE simulations were conducted for seven distinct PSC2 structures, with layer heights ranging from 7 mm to 13 mm at intervals of 1 mm. Each model, characterized by a thickness of 0.5 mm and a septa thickness of 2 mm, was subjected to compression up to 75% of its original height. 

**Figure 10:**
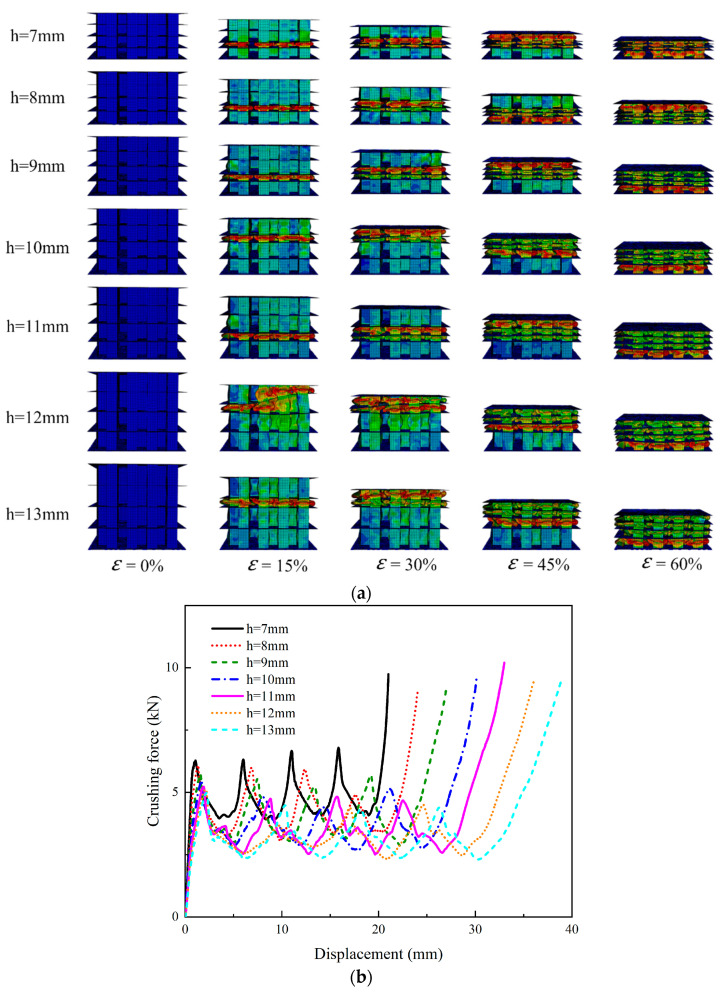
Numerical simulation results for the PSC2 models with different layer heights: (**a**) deformation modes; (**b**) crushing-force–displacement curves.

**Figure 11:**
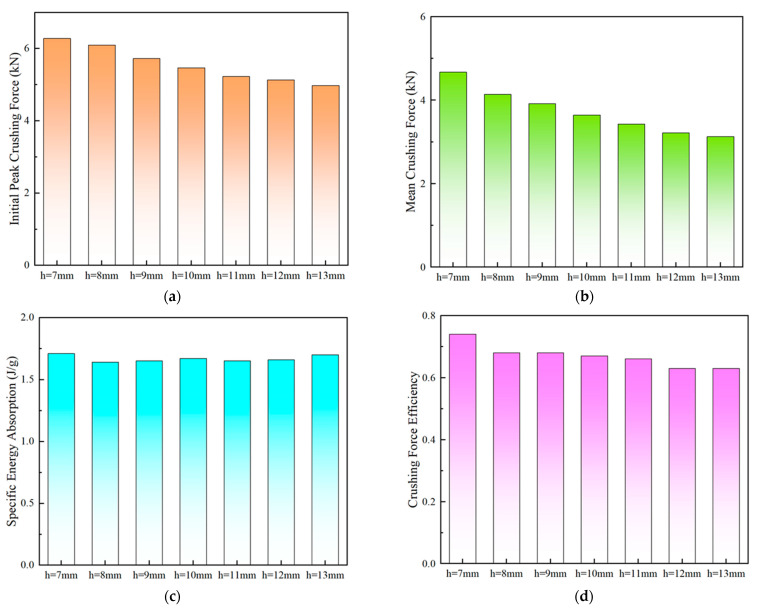
Energy absorption performance parameters with respect to layer height: (**a**) *IPF*; (**b**) *MCF*; (**c**) *SEA*; (**d**) *CFE*.

Figure 10a shows the numerical simulation results, and it was clearly found that PSC2 models with different layer heights had almost the same deformation modes. Figure 10b illustrates the crushing-force–displacement curves of the PSC2 model under quasi-static axial crushing conditions with varying layer heights. It was evident that all curves exhibited a similar trend, with each curve having four peaks and the peak force gradually decreasing as the layer height increased. The model with a layer height of 7 mm exhibited a higher resistance to the impact load than other samples. The energy absorption characteristic parameters are summarized in Table 3 and plotted in Figure 11. As shown in Figure 11a,b, both the *IPF* and *MCF* showed a trend of gradually decreasing with the increase in the layer height, indicating that the PCS2 structure could resist a higher impact force at a lower layer height. In Figure 11c, it can be observed that the *SEA* values for different layer heights were similar. Figure 11d illustrates a general decreasing trend in the *CFE*. Notably, the structure with a layer height of 7 mm exhibited the highest *CFE*, which was significantly greater than that of the other samples. The *CFE* of PSC2 with an h of 8 mm only experienced an 8.1% decrease compared to PSC2 with an h of 7 mm. However, when the layer height was increased to 13 mm, the *CFE* decreased by 15.8% compared to the case with an h of 7 mm.

Based on the aforementioned results, it could be concluded that the energy absorption performance of the PCS2 model could be improved by appropriately reducing the height of the layer.

#### 4.2.2. Effect of Septa Thickness

The sandwich septa of the PSC2 structure function to damp and buffer the crushing force of thin-walled structures. This section primarily investigates the effect of the septa thickness d on the crashworthiness of the PSC2 structure. The values of d tested were 0.5 mm, 1 mm, 1.5 mm, 2 mm, 2.5 mm, and 3 mm. 

**Figure 12:**
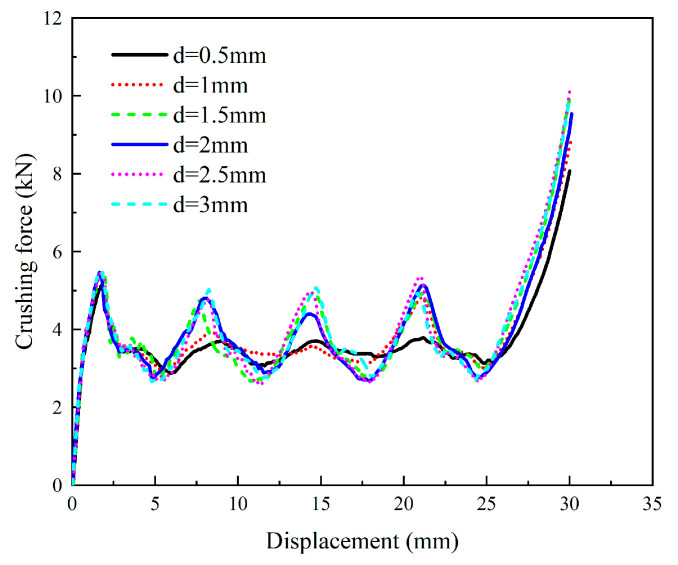
Crushing-force–displacement curves for PSC2 models with different septa thicknesses.

**Figure 13:**
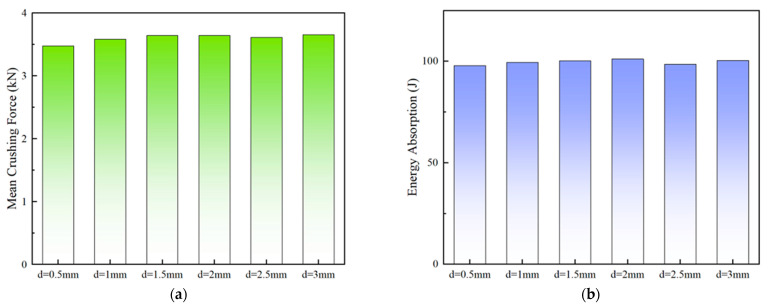

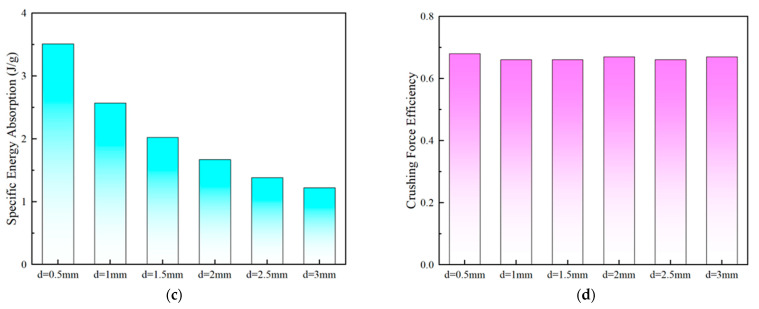
Energy absorption performance parameters with respect to septa thickness: (**a**) *IPF*; (**b**) *MCF*; (**c**) *SEA*; (**d**) *CFE*.

The crushing-force–displacement curves of the PSC2 (t= 0.5 mm, h=10 mm) model with different septa thicknesses are depicted in Figure 12. As shown in Figure 12, when the septa thickness exceeded 1 mm, the curves had similar trends, featuring four distinct peaks that represent the sequential crushing of each layer of the PSC2 structure. In contrast, when the septa thickness was 0.5 mm and 1 mm, the curves only had one and two high peaks, respectively, because the septa were incapable of resisting a large enough load during the compression process of the PSC2 structure, causing it to bend. Meanwhile, the impacts of d on the crashworthiness performance are summarized in Table 4 and presented in Figure 13. Figure 13a demonstrates the *MCF* for different septa thicknesses. The PSC2 structure with smaller d could not resist large crushing forces, resulting in lower *MCF* values for PSC2 with a d of 0.5 mm and 1 mm compared to other samples. It is noteworthy that although the *MCF* of the PSC2 structure with d= 0.5 mm was low, its energy absorption capacity was comparable to that of other samples, as shown in Figure 13b. Figure 13c displays the *SEA* values under different d. The *SEA* of the PSC2 structure with d= 0.5 mm was significantly higher than that of other samples, reaching 3.51 J/g, which is 36.6% higher than that at d= 1 mm (2.57 J/g) and nearly three times higher than that at d= 3 mm (1.22 J/g). Figure 13d presents the *CFE* values for different thicknesses, with each sample showing similar values, indicating that d had little influence on them. Consequently, under the load-bearing capacity requirement, an appropriate reduction in d could effectively conserve materials and provide a certain level of energy dissipation.

#### 4.2.3. Effect of Wall Thickness

This section examined the crashworthiness and energy absorption capabilities of PSC2 and PSC3 structures, with an emphasis on variations in wall thicknesses. The range of wall thicknesses considered in this study was from 0.1 mm to 0.5 mm in increments of 0.1 mm. Additionally, this section compares the mechanical properties of PSC2 and PSC3 structures with equivalent relative densities.

**Figure 14:**
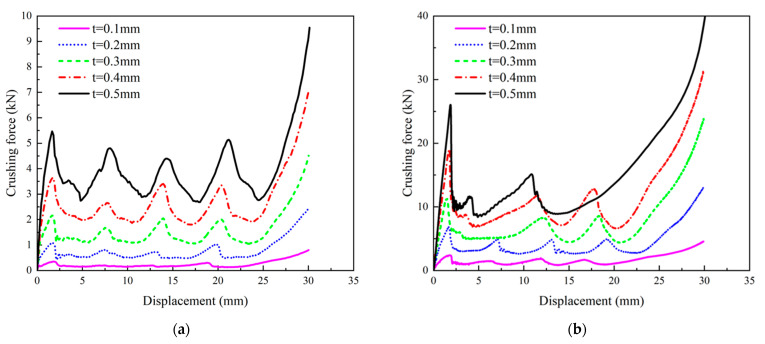
Crushing-force–displacement curves for PSC models with different wall thicknesses: (**a**) PSC2 models; (**b**) PSC3 models.

**Figure 15:**
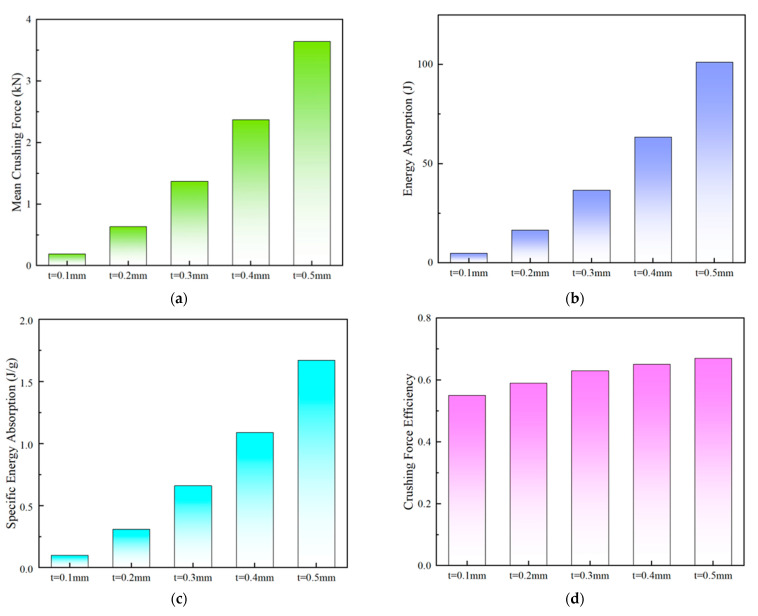
Energy absorption performance parameters of PSC2 models on wall thickness: (**a**) *MCF*; (**b**) *EA*; (**c**) *SEA*; (**d**) *CFE*.

**Figure 16:**
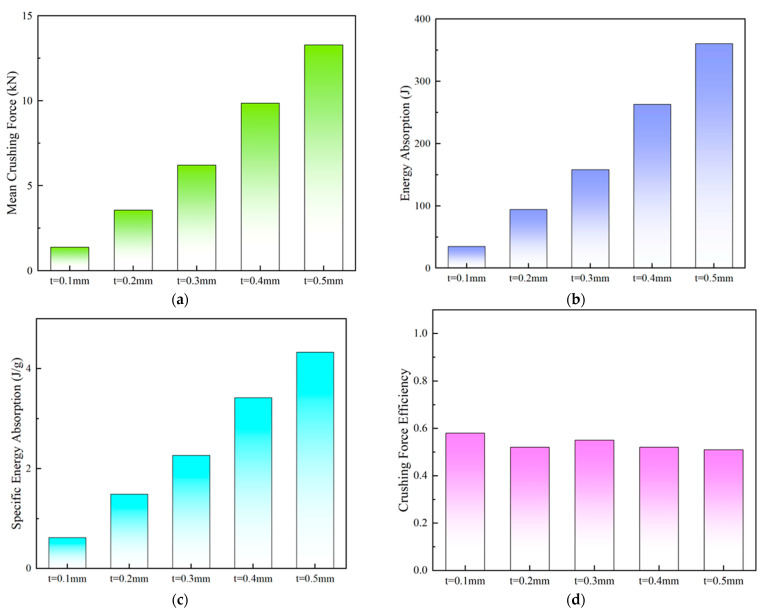
Energy absorption performance parameters of PSC3 models on wall thickness: (**a**) *MCF*; (**b**) *EA*; (**c**) *SEA*; (**d**) *CFE*.

The deformation behavior of the PSC2 model with a wall thickness ranging from 0.1 mm to 0.5 mm was found to be similar under compression. The displacement–force curves of the PSC2 model exhibited similar trends, with a notable increase in the force level as the wall thickness increased, as illustrated in Figure 14a. In contrast, the deformation behavior of the PSC3 model depended on the wall thickness. When the wall thickness of the PSC3 model was small (t≤ 0.2 mm), the curve had four peaks, as shown in Figure 14b. With an increase in wall thickness, the initial peak force increased significantly, followed by 1–2 smaller peaks, which were attributed to the deformation of the PSC3 model under compression. The sandwich septa were unable to absorb the deformation of the thin-walled structure at a larger wall thickness, resulting in the simultaneous deformation of multiple layers of the PSC3 model and an increase in the initial peak force. The crashworthiness indicators of PSC2 and PSC3 for different wall thicknesses are presented in Table 5 and depicted in Figure 15 and Figure 16. The relationships between the *MCF*, *EA*, *SEA*, and wall thickness were analyzed, and it was found that these indicators increased at a higher gradient with an increase in thickness, as shown in Figure 15a–c and Figure 16a–c. Notably, the *CFE* values of the two structures exhibited different trends. The *CFE* values of the PSC2 model gradually increased with an increase in wall thickness, as shown in Figure 15d. However, the *CFE* value of the PSC3 model was higher for smaller wall thicknesses, as shown in Figure 16d.

**Figure 17:**
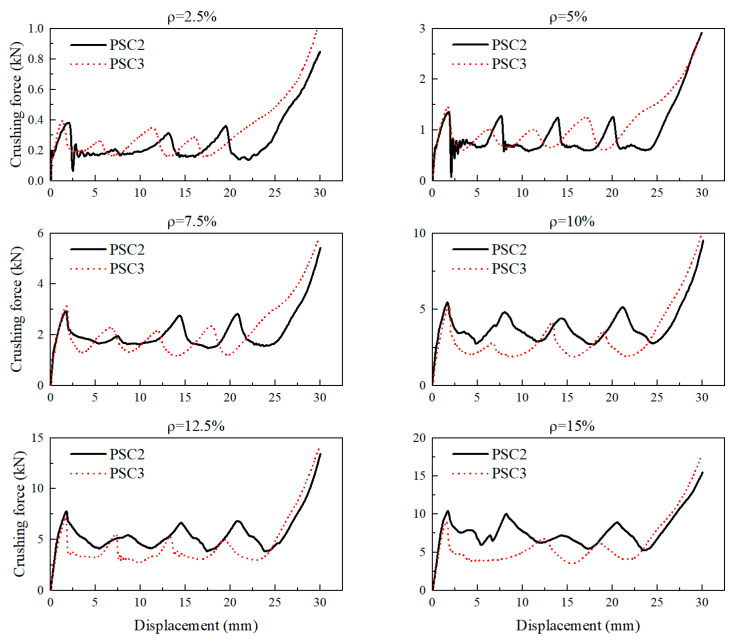
Crushing-force–displacement curves for PSC2 and PSC3 models with different relative densities.

To investigate the crashworthiness of PSC2 and PSC3 structures at the same relative density, the mechanical properties of six pairs of structures with relative densities ranging from 2.5% to 15% at intervals of 2.5% were compared after compression. The relative density, ρ, was defined as the volume fraction of a thin-walled structure with a side length of 50 mm in each layer of the model in a cube space with a side length of 50 mm and a height of 10 mm. Varying the wall thickness enabled the production of structures with different relative densities. Figure 17 shows the force–displacement curves of PSC2 and PSC3 models with the six relative densities. It can be observed in Figure 17 that the curves of the two models were similar at ρ= 2.5% and ρ= 5%. Notably, for relative densities greater than 5%, the PSC2 model had a stronger bearing capacity than the PSC3 model. Furthermore, as the relative density increased, the gap between the two structures’ crashworthiness also increased.

**Figure 18:**
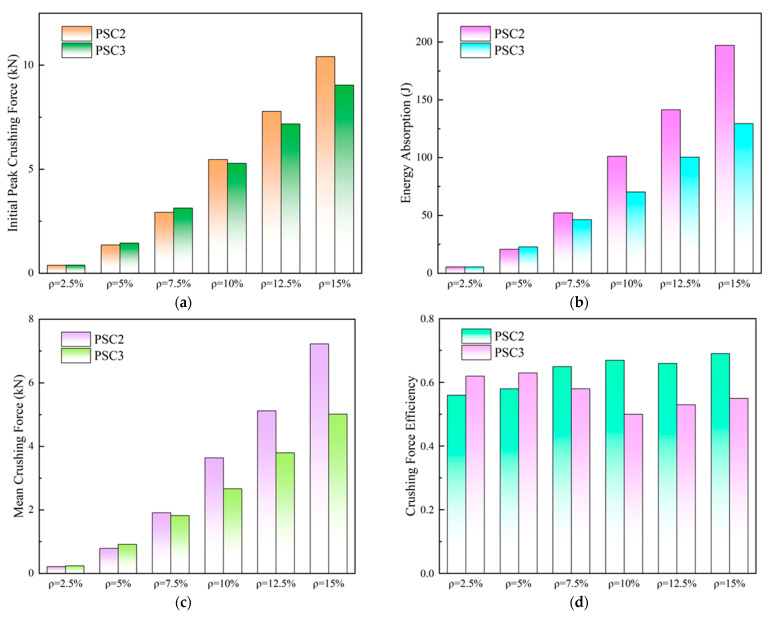
Energy absorption performance parameters of PSC2 and PSC3 models on relative density: (**a**) *IPF*; (**b**) *EA*; (**c**) *MCF*; (**d**) *CFE*.

Meanwhile, the crashworthiness performance of the two structures is presented in Figure 18 and summarized in Table 6. Figure 18a illustrates the change in the *IPF* values of the two structures with the relative density. When the relative density was lower than 7.5%, the *IPF* of the PSC3 model was slightly higher than that of the PSC2 model, while the *IPF* of the PSC2 model was higher than that of the PSC3 model when the relative density was higher. Figure 18b,c show similar variations in *EA* and *MCF* values with the relative density. When ρ did not exceed 5%, the value of the PSC3 model was slightly higher than that of PSC2. However, when the relative density increased to 7.5% and higher, the value of PSC2 was significantly higher than that of PSC3. As for *CFE*, it can be observed in Figure 18d that when ρ was small (ρ≤ 5%), the *CFE* of the PSC3 model was higher than that of PSC2. In other cases, the value of the PSC2 model was higher than that of PSC3. In addition, the *CFE* value of the PSC2 model showed an overall upward trend, while that of PSC3 showed a downward trend. 

It is worth noting that the PSC3 structure could enhance the structural durability to some extent. However, complex structures in engineering applications require more precise manufacturing processes, which increase the cost of sample preparation. Of course, this is also a significant challenge faced by many new structural applications. Therefore, conducting the in-depth validation of structures in specific environmental conditions is essential to ensure the suitability of the structural design.

## 5. Optimization and Application

### 5.1. Structural Optimization

In order to obtain multilayer thin-walled sandwich structures with optimal energy absorption characteristics, typically, structures need to have higher *SEA* while keeping the *IPF* as low as possible. Therefore, *IPF* and *SEA* were taken as the optimization targets of PSC structures, and the mathematical expressions describing the optimization problem are as follows.

PSC2:

(5) $$\left\{\begin{array}{c} Min\;\;\;\;\;\left\{IPF\left(h,\;t\right),-SEA(h,t)\}\right.\\ \;\;\;\;\;\;\;\;\;\;\;\;s.t.\;\;\;\;\;\;7\leq h\leq 12\\ \;\;\;\;\;\;\;\;\;\;\;\;\;\;\;\;\;0.125\leq t\leq 0.75 \end{array}\;\;\right.\;\;\;\;\;\;\;$$

PSC3:

(6) $$\left\{\begin{array}{c} Min\;\;\;\;\;\left\{IPF\left(h,\;t\right),-SEA(h,t)\}\right.\\ \;\;\;\;\;\;\;\;\;\;\;\;s.t.\;\;\;\;\;\;7\leq h\leq 12\\ \;\;\;\;\;\;\;\;\;\;\;\;\;\;\;\;\;0.0446\leq t\leq 0.2679 \end{array}\right.\;\;\;\;\;\;\;$$

The response surface method (RSM) is an effective approximation approach to finding the optimal process parameters by regression equation analysis and has been widely used in structural impact analysis [21,66]. Thirty-six groups of samples were obtained through numerical simulations, which are presented in Table A1 and Table A2. The approximate models of *IPF* and *SEA* were established by using RSM. The response surface models established by *IPF* and *SEA* are as follows.

PSC2:

(7) $$\begin{array}{cc} IPF=f\left(h,t\right)= & 1236.756-678.953h-36.61t-25.435ht+147.543h^{2}\\ & -93.44t^{2}-4.11h^{2}t-1.11ht^{2}-15.88h^{3}+358.328t^{3}\\ & +4.75h^{2}t^{2}+0.18h^{3}t-84.626ht^{3}+0.847h^{4}+44.91t^{4}\\ & -0.386h^{3}t^{2}+6.29h^{2}t^{3}+0.0016h^{4}t-25.219ht^{4}-0.018h^{5}\\ & +96.58t^{5} \end{array}$$

(8) $$\begin{array}{cc} SEA=f\left(h,t\right)= & 776.233-420.394h-117.88t+62.38ht+90.044h^{2}\\ & -157.04t^{2}-11.41h^{2}t+38.70ht^{2}-9.54h^{3}+146.278t^{3}\\ & -3.38h^{2}t^{2}+0.886h^{3}t-14.535ht^{3}+0.50h^{4}-113.776t^{4}\\ & +0.096h^{3}t^{2}+0.644h^{2}t^{3}-0.025h^{4}t+1.629ht^{4}-0.01h^{5}\\ & +48.88t^{5} \end{array}$$

PSC3:

(9) $$\begin{array}{cc} IPF=f\left(h,t\right)= & 148.26-74.01h-561.49t+289.83ht+14.28h^{2}\\ & -1265.74t^{2}-53.28h^{2}t+443.61ht^{2}-1.325h^{3}\\ & +792.168t^{3}-58.022h^{2}t^{2}+4.307h^{3}t+458.737ht^{3}\\ & +0.058h^{4}-9794.258t^{4}+2.232h^{3}t^{2}-9.10h^{2}t^{3}\\ & -0.128h^{4}t-675.80ht^{4}-0.001h^{5}+20555.74t^{5} \end{array}$$

(10) $$\begin{array}{cc} SEA=f\left(h,t\right)= & 103.96-53.50h-309.62t+119.79ht+10.95h^{2}\\ & +489.636t^{2}-18.642h^{2}t-20.98ht^{2}-1.11h^{3}\\ & -2698.247t^{3}-0.064h^{2}t^{2}+1.314h^{3}t+11.684ht^{3}\\ & +0.056h^{4}+9666.83t^{4}+0.198h^{3}t^{2}-10.597h^{2}t^{3}\\ & -0.036h^{4}t+342.56ht^{4}-0.001h^{5}-17566.256t^{5} \end{array}$$

In order to verify the accuracy of the response surface models, the coefficients of multiple determination ($R^{2}$) and root-mean-square error (RMSE) were used for evaluation, given by [67]:

(11) $$R^{2}=\frac{\sum_{i=1}^{N}{\left(y_{i}-\bar{y}\right)}^{2}-{\left(y_{i}-y_{i}^{\prime }\right)}^{2}}{\sum_{i=1}^{N}{\left(y_{i}-\bar{y}\right)}^{2}}\;\;\;\;\;$$

(12) $$RMSE=\sqrt{\frac{\sum_{i=1}^{N}{\left(y_{i}-y_{i}^{\prime }\right)}^{2}}{N-1}}\;\;$$

where $y_{i}$ and $y_{i}^{\prime }$ are the simulation value of the sample point and the corresponding value of the response surface model, respectively. $\bar{y}$ is the mean value of all sample points, and N is the number of sample points.

**Figure 19:**
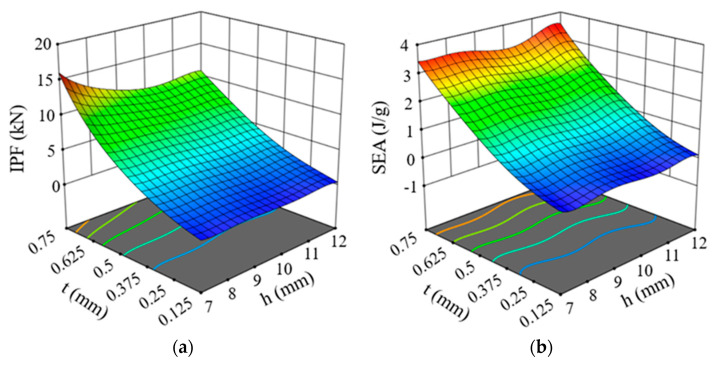

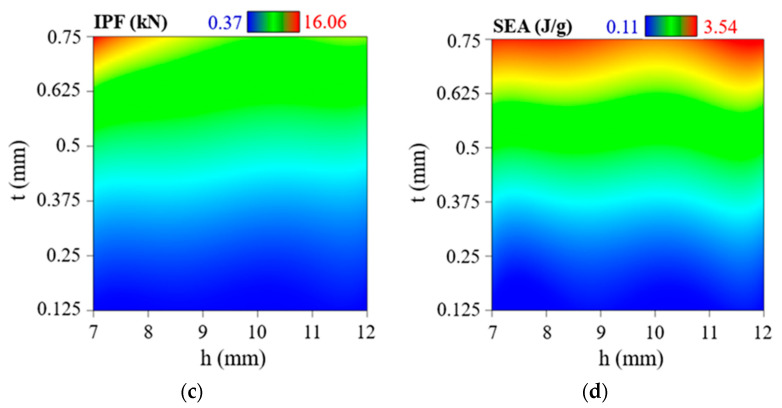
Response surface models of PSC2 structure: (**a**) 3D surface of *IPF*; (**b**) 3D surface of *SEA*; (**c**) contour map of *IPF*; (**d**) contour map of *SEA*.

**Figure 20:**
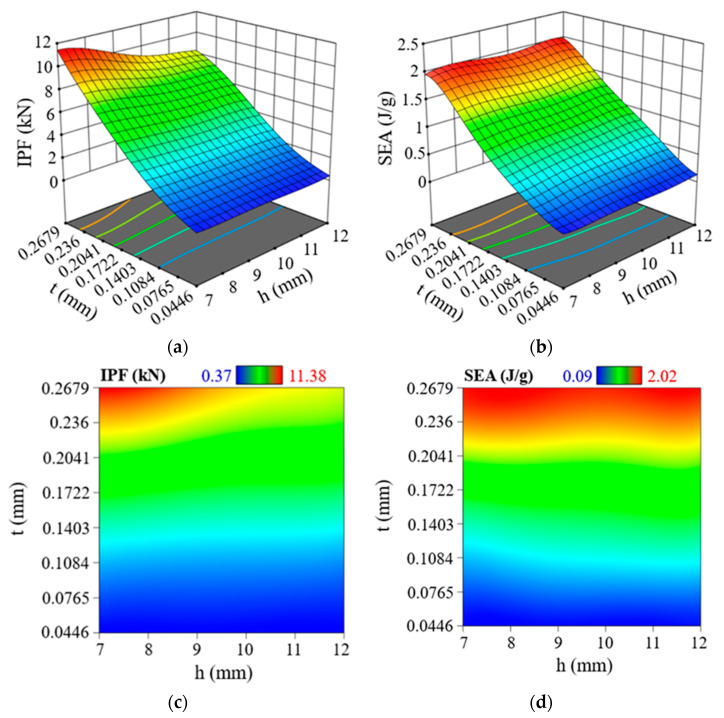
Response surface models of PSC3 structure: (**a**) 3D surface of *IPF*; (**b**) 3D surface of *SEA*; (**c**) contour map of *IPF*; (**d**) contour map of *SEA*.

$R^{2}$ indicates how closely the regression predictions match the data points obtained from the finite element (FE) simulation, while RMSE is commonly used to estimate the overall accuracy of the model. In general, as $R^{2}$ approaches 1, the RMSE becomes smaller, indicating a higher level of model accuracy. The PSC2 model produced a fitted function of *IPF* with $R^{2}$ and RMSE values of 0.9997 and 0.074, respectively, while the fitted function of *SEA* had $R^{2}$ and RMSE of 0.9985 and 0.045, respectively. For the PSC3 model, the values of $R^{2}$ and RMSE for the fitted function of *IPF* were 0.9998 and 0.046, respectively, and the fitted function of *SEA* had $R^{2}$ and RMSE values of 0.9995 and 0.015, respectively. Therefore, the established response surface model had sufficient accuracy. The response surface function diagrams and contour diagrams of the PSC2 model and PSC3 model are shown in Figure 19 and Figure 20, respectively. It can be seen from Figure 19a,c that the *IPF* of the PSC2 model increased with the increase in wall thickness or the decrease in layer height. Figure 19b shows the change in *SEA* with the wall thickness and layer height. With the increase in t, *SEA* increased, while h had little effect on *SEA* (see Figure 19d). Similar to the PSC2 model, the *IPF* of the PSC3 model also increased with the increase in t and the decrease in h, but the influence of wall thickness was more significant, as shown in Figure 20a,c. Figure 20b reveals that when increasing t, the *SEA* value was increased. However, the change in h had nearly no effect on the *SEA* value (see Figure 20d).

**Figure 21:**
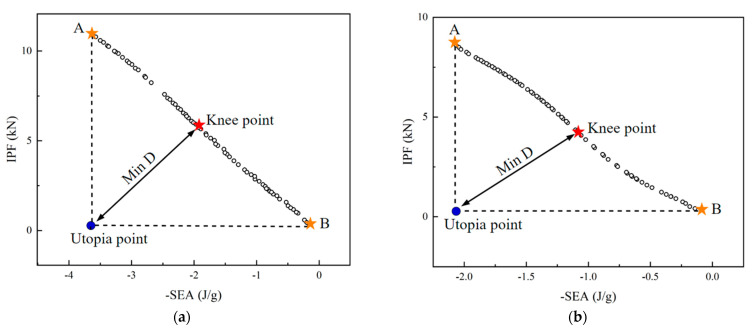
Pareto front of the two structures: (**a**) PSC2 structure; (**b**) PSC3 structure.

Furthermore, the NSGA-II algorithm was used to optimize the *IPF* and *SEA* of the two models, and the Pareto solution set obtained is shown in Figure 21. Any point located on the Pareto boundary could be regarded as an optimal solution, but in practical engineering, engineers can choose the optimal structure according to different constraints. For example, when the structure was designed to maximize the energy absorption capacity, optimal point A was obtained. On the other hand, if the focus was solely on minimizing the impact acceleration, optimal point B was identified, where the *IPF* was minimized. In this study, to achieve a balance between the *SEA* and *IPF* indices, the minimum distance selection method (TMDSM) was employed to identify a knee point on the Pareto front, which can be formulated by [21,68]:

(13) $$minD=\sqrt{\frac{1}{K}\sum_{i=1}^{K}{\left(\frac{f_{ci}-\min\left(f_{i}\left(x\right)\right)}{\max\left(f_{i}\left(x\right)\right)-\min\left(f_{i}\left(x\right)\right)}\right)}^{2}}$$

where K is the number of objective components, $f_{ci}$ is the ith objective value in the cth Pareto solution, and D represents the distance from the “Utopia point” to a point on the Pareto curve.

A comparison of optimization and numerical simulation results is shown in Table 7. The errors of both structures were acceptable, which indicates that the optimization results were accurate enough.

### 5.2. Effect of Material Choice

The use of different materials may significantly affect the performance of thin-walled structures. In the previous part of this study, Tough 1500 Resin was employed for the multilayer thin-walled sandwich structures. However, in the context of energy-absorbing materials for thin-walled structures, aluminum alloy and stainless steel have frequently been chosen due to their lightweight nature and high resistance to crushing [69,70]. Recognizing this, the present study investigated the influence of AA6061 aluminum alloy and 316L stainless steel on the crushing responses of multilayer thin-walled sandwich structures. The material properties utilized for numerical simulations are presented in Table 8.

**Figure 22:**
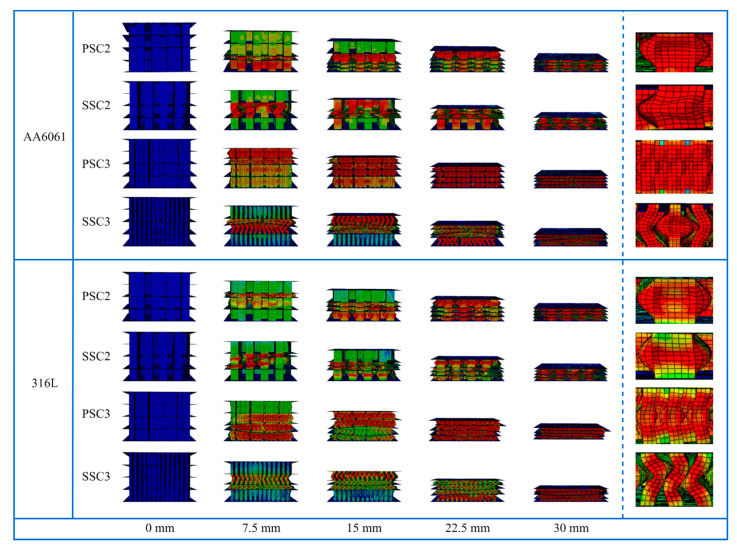
Numerical simulation results of four models with AA6061 and 316L materials.

**Figure 23:**
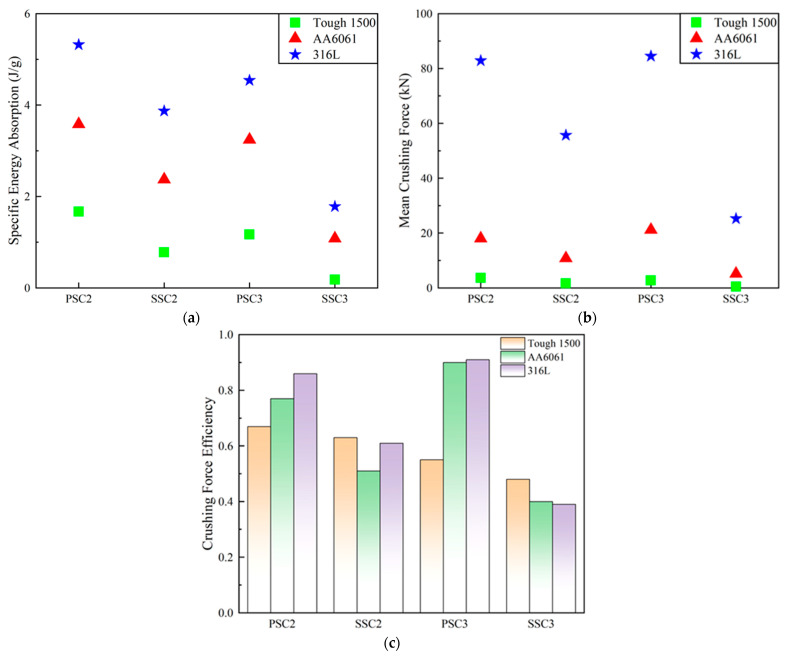
Energy absorption performance parameters of four models with different materials: (**a**) *SEA*; (**b**) *MCF*; (**c**) *CFE*.

The quasi-static compression simulation of the four models was conducted using the material properties of AA6061 and 316L, and the crushing process is visualized in Figure 22. The deformation of each model, when simulated with these two metal materials, exhibited significant improvement, with no compression instability observed in any of the models. It is worth noting that the PSC3 structure displayed a deformation mode similar to that of the PSC2 structure, characterized by outward expansion in the middle of the thin wall, which notably enhanced its energy absorption capabilities. Figure 23 illustrates the *SEA*, *MCF*, and *CFE* of the four models with the different materials. Previous numerical simulation results indicated that, when using Tough 1500 for all four models, the PSC2 structure exhibited the best crashworthiness. Similarly, when considering the other three materials, the PSC2 structure continued to have the highest *SEA*, as shown in Figure 23a. However, it is evident from Figure 23b,c that the *MCF* and *CFE* values of the PSC3 structure saw significant improvements with AA6061 and 316L materials, surpassing those of the other structures. It is particularly noteworthy that, when using AA6061 and 316L, the PSC3 structure achieved exceptionally high crushing force efficiencies of 0.90 and 0.91, respectively, approaching the performance of an ideal energy absorber.

### 5.3. Application

**Figure 24:**
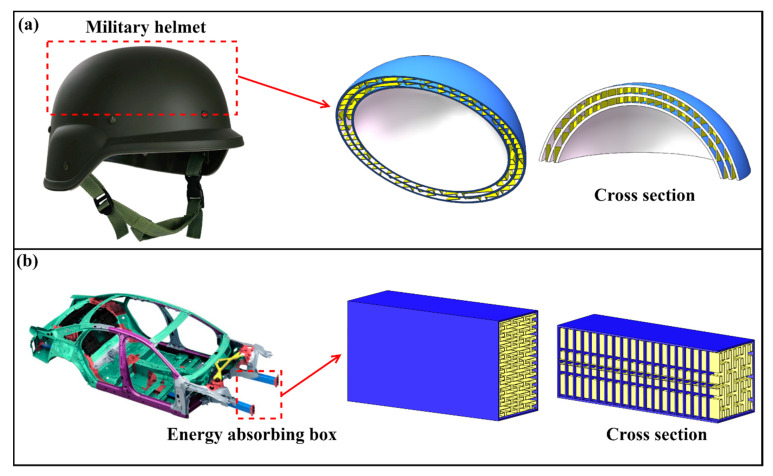
Application of PSC structure in (**a**) military helmet and (**b**) energy-absorbing box [71].

Based on the aforementioned research, it can be observed that PSC structures exhibited a high level of impact resistance and energy absorption capability, making them promising for various engineering applications. Additionally, the PSC structures could be further optimized based on specific application requirements, as shown in Figure 24. 

PSC structures could be employed in the design of military helmets, as illustrated in Figure 24a. Military helmets play a crucial role in safeguarding the heads of soldiers during impact events. Helmets designed with PSC structures could absorb a significant portion of the energy during impact, and the incorporation of internal voids and thin walls in the structure serves to reduce the helmet’s weight, making it more convenient for portability. Additionally, the PSC structure could be employed in the design of automotive energy-absorbing boxes [71]. When a car experiences a collision, the energy-absorbing box must absorb a significant portion of the energy generated by the impact. The multilayered sandwich metal energy-absorbing box constructed based on the PSC structure is depicted in Figure 24b. It exhibited excellent impact resistance and energy absorption capabilities, ensuring passenger safety. The matrix material used in the PSC structure should be selected and verified according to the application environment in order to make the quantification of mechanical properties more appropriate.

## 6. Conclusions

In this paper, novel multilayer thin-walled sandwich energy absorption structures are proposed based on the PSC to achieve ideal energy absorption. Polymer specimens were fabricated using 3D printing technology, and their compression behaviors and energy absorption properties were investigated through tests and FE simulations. The results indicated that PSC structures exhibited superior energy absorption performance compared to SSC structures with the same mass. Furthermore, the effects of layer height, septa thickness, and wall thickness on the crashworthiness of the PSC structure were studied through parametric analysis, which provided a reference for the selection of structural parameters in practical applications. Finally, the response surface method and the NSGA-II algorithm were used to further optimize the structure, and the effects of different materials on the mechanical properties of the structure were compared. The potential application of the PSC structure in the engineering field was also presented. In the future, with the further development of 3D printing technology, the difficulty and cost of preparing complex structures will also be reduced.

In summary, the novel multilayer thin-walled sandwich energy absorption structures proposed in this paper represent a novel approach to designing lightweight polymer structures with exceptional energy-absorbing properties, thereby offering significant potential for engineering applications.

## Figures and Tables

**Table 1:** Crashworthiness data of experiment and simulation.

Type	Mass (g)	*SEA* (J/g)	*MCF* (kN)
PSC2 (Exp.1)	60.16	1.68	3.60
PSC2 (Exp.2)	60.60	1.73	3.69
PSC2 (Sim)	60.50	1.67	3.64
SSC2 (Exp.1)	59.86	0.62	1.47
SSC2 (Exp.2)	60.03	0.72	1.66
SSC2 (Sim)	60.50	0.78	1.67

**Table 2:** Crashworthiness indicators of four models.

Type	Mass (g)	*IPF* (kN)	*EA* (J)	*SEA* (J/g)	*MCF* (kN)	*CFE*
PSC2	60.50	5.47	101.22	1.67	3.64	0.67
SSC2	60.50	2.67	47.34	0.78	1.67	0.63
PSC3	60.50	4.94	70.74	1.17	2.72	0.55
SSC3	60.50	0.93	11.10	0.18	0.45	0.48

**Table 3:** Energy-absorbing characteristics of PSC2 models with different layer heights.

h (mm)	Mass (g)	*IPF* (kN)	*EA* (J)	*SEA* (J/g)	*MCF* (kN)	*CFE*
7	56.72	6.28	96.05	1.71	4.67	0.74
8	57.98	6.09	94.96	1.64	4.14	0.68
9	59.24	5.72	97.89	1.65	3.92	0.68
10	60.50	5.47	101.22	1.67	3.46	0.67
11	61.76	5.23	101.63	1.65	3.43	0.66
12	63.02	5.13	104.78	1.66	3.22	0.63
13	64.28	4.98	109.08	1.70	3.13	0.63

**Table 4:** Energy-absorbing characteristics of PSC2 models with different septa thicknesses.

d (mm)	Mass (g)	*IPF* (kN)	*EA* (J)	*SEA* (J/g)	*MCF* (kN)	*CFE*
0.5	27.83	5.12	97.80	3.51	3.48	0.68
1	38.72	5.39	99.49	2.57	3.58	0.66
1.5	49.61	5.48	100.22	2.02	3.64	0.66
2	60.50	5.47	101.22	1.67	3.64	0.67
2.5	71.39	5.50	98.55	1.38	3.61	0.66
3	82.28	5.48	100.34	1.22	3.65	0.67

**Table 5:** Energy-absorbing characteristics of PSC2 and PSC3 models with different wall thicknesses.

Type	t (mm)	Mass (g)	*IPF* (kN)	*EA* (J)	*SEA* (J/g)	*MCF* (kN)	*CFE*
PSC2	0.1	50.82	0.35	4.90	0.10	0.19	0.55
	0.2	53.24	1.08	16.54	0.31	0.63	0.59
	0.3	55.66	2.16	36.70	0.66	1.37	0.63
	0.4	58.08	3.65	63.40	1.09	2.37	0.65
	0.5	60.50	5.47	101.22	1.67	3.64	0.67
PSC3	0.1	56.05	2.40	34.80	0.62	1.38	0.58
	0.2	62.82	6.84	93.89	1.49	3.56	0.52
	0.3	69.60	11.25	158.06	2.27	6.21	0.55
	0.4	76.38	19.07	262.73	3.42	9.85	0.52
	0.5	83.15	26.04	360.26	4.33	13.28	0.51

**Table 6:** Energy-absorbing characteristics of PSC2 and PSC3 models with different relative densities.

Type	ρ	t (mm)	Mass (g)	*IPF* (kN)	*EA* (J)	*SEA* (J/g)	*MCF* (kN)	*CFE*
PSC2	2.5%	0.1250	51.06	0.38	5.47	0.11	0.21	0.56
	5%	0.2500	54.21	1.36	20.81	0.38	0.79	0.58
	7.5%	0.3750	57.35	2.93	52.16	0.91	1.91	0.65
	10%	0.5000	60.50	5.47	101.22	1.67	3.64	0.67
	12.5%	0.6250	63.65	7.78	141.47	2.22	5.12	0.66
	15%	0.7500	66.79	10.41	197.15	2.95	7.22	0.69
PSC3	2.5%	0.0446	51.06	0.39	5.56	0.11	0.24	0.62
	5%	0.0893	54.21	1.45	22.75	0.42	0.92	0.63
	7.5%	0.1339	57.35	3.13	46.41	0.81	1.82	0.58
	10%	0.1786	60.50	5.28	70.33	1.16	2.66	0.50
	12.5%	0.2232	63.65	7.18	100.38	1.58	3.80	0.53
	15%	0.2679	66.79	9.04	129.46	1.94	5.02	0.55

**Table 7:** The validation of optimization solutions and numerical results for PSC2 and PSC3 structures.

Type	Case	h (mm)	t (mm)	*IPF* (kN)			*SEA* (J/g)		
				Opt Result	Num Result	Er (%)	Opt Result	Num Result	Er (%)
PSC2	Point A	12	0.75	10.88	10.89	0.092	3.56	3.54	0.565
	Point B	12	0.125	0.32	0.36	11.111	0.128	0.125	2.400
	Knee point	11.8	0.525	5.74	5.65	1.593	1.93	1.82	6.044
PSC3	Point A	11.6	0.2679	8.60	8.38	2.625	2.06	2.01	2.488
	Point B	8	0.0446	0.39	0.41	4.878	0.091	0.090	1.111
	Knee point	11.9	0.1607	4.29	4.07	5.405	1.09	1.01	7.92

**Table 8:** Materials properties for numerical simulations.

Materials	Density $\left(\mathrm{kg}/m^{3}\right)$	Young’s Modulus (MPa)	Poisson Ratio	Yield Strength (MPa)	Ultimate Strength (MPa)	Ref.
Tough 1500	1210	850	0.35	48.6	58.51	This study
AA6061	2700	68,000	0.33	71.0	130.70	[69]
316L	7980	190,600	0.30	380.0	821.30	[70]

## Data Availability

The data presented in this study are available on request from the corresponding author.

## Appendix A

**Table A1:** Sample points and numerical simulation results of PSC2 model.

No.	h (mm)	t (mm)	*IPF* (kN)	*SEA* (J/g)
1	7	0.125	0.49	0.108
2	7	0.25	1.76	0.42
3	7	0.375	3.78	0.95
4	7	0.5	6.28	1.69
5	7	0.625	10.19	2.48
6	7	0.75	16.06	343
7	8	0.125	0.45	0.112
8	8	0.25	1.61	0.4
9	8	0.375	3.52	0.92
10	8	0.5	6.09	1.64
11	8	0.625	8.83	2.48
12	8	0.75	12.85	3.39
13	9	0.125	0.43	0.111
14	9	0.25	1.52	0.41
15	9	0.375	3.30	0.95
16	9	0.5	5.72	1.65
17	9	0.625	8.47	2.5
18	9	0.75	11.41	3.37
19	10	0.125	0.38	0.11
20	10	0.25	1.36	0.39
21	10	0.375	2.93	0.92
22	10	0.5	5.47	1.67
23	10	0.625	7.78	2.24
24	10	0.75	10.41	2.98
25	11	0.125	0.39	0.12
26	11	0.25	1.41	0.43
27	11	0.375	3.07	0.97
28	11	0.5	5.23	1.65
29	11	0.625	8.13	2.49
30	11	0.75	10.67	3.45
31	12	0.125	0.37	0.125
32	12	0.25	1.34	0.43
33	12	0.375	2.97	0.98
34	12	0.5	5.13	1.66
35	12	0.625	7.81	2.51
36	12	0.75	10.89	3.54

**Table A2:** Sample points and numerical simulation results of PSC3 model.

No.	h (mm)	t (mm)	*IPF* (kN)	*SEA* (J/g)
1	7	0.0446	0.44	0.109
2	7	0.0893	1.69	0.32
3	7	0.1339	3.50	0.66
4	7	0.1786	5.79	1.10
5	7	0.2232	8.50	1.63
6	7	0.2679	11.38	1.96
7	8	0.0446	0.41	0.09
8	8	0.0893	1.55	0.40
9	8	0.1339	3.31	0.71
10	8	0.1786	5.55	1.11
11	8	0.2232	8.11	1.69
12	8	0.2679	10.96	2.02
13	9	0.0446	0.40	0.101
14	9	0.0893	1.49	0.42
15	9	0.1339	3.23	0.79
16	9	0.1786	5.38	1.16
17	9	0.2232	7.50	1.58
18	9	0.2679	9.95	1.99
19	10	0.0446	0.39	0.108
20	10	0.0893	1.45	0.42
21	10	0.1339	3.13	0.81
22	10	0.1786	5.28	1.16
23	10	0.2232	7.18	1.58
24	10	0.2679	9.04	1.94
25	11	0.0446	0.39	0.121
26	11	0.0893	1.40	0.42
27	11	0.1339	3.02	0.83
28	11	0.1786	5.16	1.20
29	11	0.2232	7.17	1.64
30	11	0.2679	8.66	2.00
31	12	0.0446	0.37	0.124
32	12	0.0893	1.39	0.46
33	12	0.1339	2.94	0.81
34	12	0.1786	4.91	1.18
35	12	0.2232	7.00	1.61
36	12	0.2679	8.31	1.99
